# Frequency and amplitude modulation of ultra-compact terahertz quantum cascade lasers using an integrated avalanche diode oscillator

**DOI:** 10.1038/srep23053

**Published:** 2016-03-15

**Authors:** Fabrizio Castellano, Lianhe Li, Edmund H. Linfield, A. Giles Davies, Miriam S. Vitiello

**Affiliations:** 1 NEST, CNR - Istituto Nanoscienze and Scuola Normale Superiore, Piazza San Silvestro 12, 56127, Pisa, Italy; 2 School of Electronic and Electrical Engineering, University of Leeds, Leeds LS2 9JT, UK

## Abstract

Mode-locked comb sources operating at optical frequencies underpin applications ranging from spectroscopy and ultrafast physics, through to absolute frequency measurements and atomic clocks. Extending their operation into the terahertz frequency range would greatly benefit from the availability of compact semiconductor-based sources. However, the development of any compact mode-locked THz laser, which itself is inherently a frequency comb, has yet to be achieved without the use of an external stimulus. High-power, electrically pumped quantum cascade lasers (QCLs) have recently emerged as a promising solution, owing to their octave spanning bandwidths, the ability to achieve group-velocity dispersion compensation and the possibility of obtaining active mode-locking. Here, we propose an unprecedented compact architecture to induce both frequency and amplitude self-modulation in a THz QCL. By engineering a microwave avalanche oscillator into the laser cavity, which provides a 10 GHz self-modulation of the bias current and output power, we demonstrate multimode laser emission centered around 3 THz, with distinct multiple sidebands. The resulting microwave amplitude and frequency self-modulation of THz QCLs opens up intriguing perspectives, for engineering integrated self-mode-locked THz lasers, with impact in fields such as nano- and ultrafast photonics and optical metrology.

Optical frequency combs have been demonstrated to have wide scale applications in optical metrology^1^, spectroscopy^2^, and in the generation of ultra-short pulse trains^3^. The extension of such techniques to both mid-infrared and terahertz (THz) frequencies^4^,^5^ would provide a step change in molecular spectroscopy and sensing, and in the many other applications identified for these regions of the electromagnetic spectrum. But, it has been a long-standing challenge for the international research community. However, mid-infrared and THz quantum cascade lasers (QCLs)^6^ are ideally suited to realize this ambition, owing to their small size, high spectral purity^7^, and their versatility to offer large-bandwidth gain media^8^.

Traditionally, a number of different technological and optical methodologies have been used to generate optical frequency combs, including laser intra-cavity phase modulation^9^, optical pumping of micro-resonators^10^, down-conversion of a higher-frequency comb^11^, and up-conversion of a lower-frequency source^12^. The most powerful comb sources, however, are mode-locked lasers. The generation of frequency combs in QCLs usually exploits injection locking of the laser cavity, a technique in which a microwave signal is superimposed onto the laser bias in order to modulate the gain at the round-trip frequency of the cavity^13^,^14^,^15^. The resulting comb formation in QCLs follows a unique dynamic where four wave mixing, combined with the QCL’s inherent short gain recovery time, induces the laser to operate in the self-frequency-modulated regime, characterized by a constant power in the time domain and a stable coherent comb in the frequency domain^16^. However, from a technological point of view, there is an essential need to develop compact and integrated systems that do not rely on external microwave inputs for the generation of the frequency comb.

Recently, QCL-based frequency combs have been demonstrated in the THz frequency range, without the need for external seeding, by engineering the refractive index dispersion of the QCL active region^17^, or through self-seeding devices that exploit an octave-spanning emission^18^. However, in order for such systems to be employed in high-resolution spectroscopy, frequency combs need to be phase-stabilized to an absolute frequency reference. In the case of injection-locked QCLs, the microwave seed is responsible for the stability of the comb. But, when THz QCL combs are achieved by dispersion compensation or self-seeding, no such reference exists. Whilst comb operation can be determined by measuring the microwave beatnote obtained by mixing THz modes, and although the collapse of the beatnote linewidth indicates that THz modes are equally spaced, there is an essential requirement for a mechanism to phase-stabilize the THz comb.

In this letter, we propose an alternative technique to achieve self-mode-locking in THz QCLs, based on the generation of a microwave seed *within the QCL cavity itself*. A microwave oscillator is integrated into the QCL cavity in order to produce a time-dependent modulation of the optical gain. GaAs-based microwave oscillators have previously been shown to emit radiation up to 150 GHz by exploiting the negative differential resistance of avalanche-based diodes^19^,^20^,^21^,^22^. By engineering a THz frequency QCL in a double-metal waveguide with an integrated GaAs-based tunable avalanche oscillator, we report observation of the emission of frequency and amplitude modulated THz radiation. This provides an integrated solution to achieve gain modulation at the cavity round-trip frequency and, at the same time, phase locking through the stabilization of the microwave oscillator.

## Results

### Terahertz lasing

The QCL active region used in our work was based on the three-well design that has shown the highest operating temperature to date^23^. The gain medium was grown in two variants: a reference structure (A), incorporating a 70-nm-thick n^+^ top contact layer and a 50-nm-thick n^+^ bottom contact layer, and a modified version (B) in which these contact layers were reduced to 50 nm and 30 nm, respectively. The reduction of the contact layer thickness is expected to change the electrostatics of the active region in proximity to the metal-semiconductor junction, effectively forming a diode that can be driven into the avalanche regime. The devices were processed into a standard Au-Au double metal waveguide configuration with Cr/Au (10/150 nm) metallization (See Methods). Cleaved laser stripes were indium-soldered onto copper sample holders and mounted in a closed-cycle helium-cooled cryostat. Light-current-voltage (LIV) curves were measured in pulsed mode, with the radiation collected by a pyro-electric detector placed in close proximity to the laser facet (see Methods). In the conventional double-metal forward biasing polarity, a positive bias is applied to the top contact with the electrons injected from the bottom contact.

**Figure 1:**
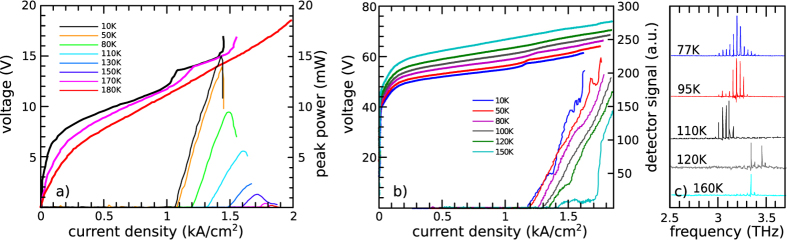
Electrical and optical characterization. (**a**,**b**) Current-voltage (thick lines, left y axis) and light-current (thin lines, right y axis) characteristics of the (**a**) reference sample A (dimensions 1200 × 180 μm), and (**b**) sample B having a 30 nm thick injection contact (dimensions 1720 × 140 μm). Being the responsivity of the pyroelectric detector unknown in the microwave regime, the vertical scale in (**b**) shows the raw voltage signal read by the pyroelectric sensor. (**c**) Emission spectra of a sample from the same batch of sample B (dimensions 1100 × 140 μm) at different heat sink temperatures. All measurements were performed in pulsed mode by driving the QCL with a 200 ns pulses and at a 10 kHz repetition rate.

Figure 1(a) shows the LIV characteristics of reference sample A. Lasing is observed up to 180 K with a low-temperature threshold current density of 1.1 kA/cm^2^, a performance consistent with the literature^23^. The LIV characteristics of sample B are shown in Fig. 1(b). The IV curve is characterized by a large threshold voltage V_T_, which increases with the heat sink temperature (T_H_). At T_H_ = 10 K, current starts to flow into the device for applied voltages larger than V_T_ ~ 40 V, beyond which the IV curve resembles that of the reference sample A. The presence of a large threshold voltage in the thin-contact sample is an indication that the metal-semiconductor junction behaves like a diode. However, at T_H_ = 10 K, the lasing threshold current density at ~1.1 kA/cm^2^ and the corresponding kink visible in the IV curve, are in agreement with the behavior of reference sample A, but with an offset in bias. The most interesting feature of sample B data is that no rollover of the emission and no region of negative differential resistance (NDR) are observed, but rather the signal increases indefinitely at higher current densities. This is in contrast to the behavior observed in sample A, which shows a visible NDR at ~1.4 kA/cm^2^ (10 K).

Figure 1(c) shows the emission spectra of sample B, demonstrating multiple mode emission centered at ~3.2 THz, which is visible up to 160 K. These measurements were performed with a Fourier transform infrared spectrometer (FTIR) equipped with a silicon bolometer. Despite the large signal measured by the pyro-electric detector shown in Fig. 1(b), it was not possible to measure any FTIR spectra of the radiation emitted by the sample at current densities larger than 1.6 kA/cm^2^. In order to exclude the possibility that the signal measured on the pyro-electric detector beyond 1.6 kA/cm^2^ resulted from electromagnetic pickup, we inserted a metallic shutter, which confirmed that this signal was radiative and could be easily removed by blanking. Any thermal emission from the QCL, arising from the large dissipated peak electrical power, was also excluded by repeating the measurement after inserting a set of infrared filters between the QCL and the detector. It was subsequently determined, as will be shown below, that the signal at high current densities was not THz radiation, but rather microwave radiation emitted by the sample.

**Figure 2:**
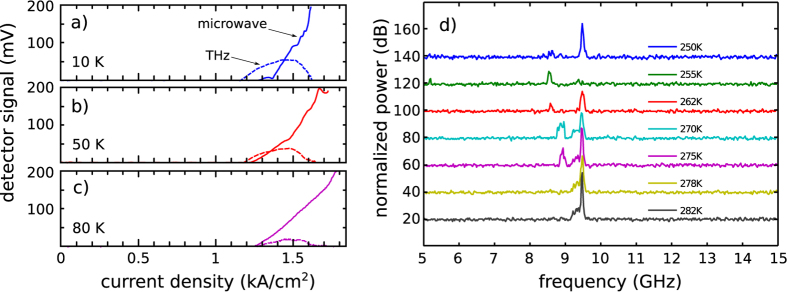
Terahertz and microwave emission. (**a**–**c**) Microwave (solid line) and THz (dashed line) components of the light-current characteristics of sample B, measured at heat sink temperatures of (**a**) 10 K, (**b**) 50 K, and (**c**) 80 K. Being the responsivity of the pyroelectric detector unknown in the microwave regime, the vertical axis shows the raw voltage signal read by the pyroelectric sensor, which is proportional to the emitted radiation intensity. (**d**) Microwave emission spectrum of a sample B collected at heat sink temperatures in the range 250–285 K. The sample was biased in pulsed mode (200 ns pulse width and 10 kHz repetition rate), and the displayed microwave power is normalized to the noise floor of the spectrum analyzer.

In order to separate the THz and microwave contributions in the light-current plot, the LIV measurements were repeated with a 2 THz low-pass filter between sample B and the pyro-electric detector. In this way, two separate LI plots can be produced, one for the THz emission alone, and one for the microwave emission. Figure 2(a–c) show the LI characteristics of sample B at T_H_ = 10 K, 50 K and 80 K, respectively, with the continuous line representing the microwave signal measured with the low-pass filter in place, and the dashed line representing the THz signal obtained as a difference between the signals measured with and without the filter. Since the THz power is obtained as a difference, and since the signal induced by the microwave emission is much stronger than the THz-frequency signal, it was not possible to separate the two signal components clearly for T_H_ > 80 K. However, the FTIR spectra shown in Fig. 1(c) unambiguously prove that the THz QCL is lasing up to T_H_ = 160 K.

Figure 2(a–c) show the expected rollover of the THz emission, as well as the increasing threshold current with temperature, but qualitative differences between the reference sample A (Fig. 1a) and sample B can still be identified. A sharp roll-off of emission is observed in sample A, which is not seen in the LIV curves of sample B (Fig. 1b). Furthermore, at 10 K, the thin-contact device (sample B) emits THz radiation over the current density range J = 1.1–1.6 kA/cm^2^, whilst in the reference sample the emission was limited to the J = 1.1–1.4 kA/cm^2^ range. Normally, when a laser is biased in the NDR, the static electric field through the active region is not uniform and a high-field domain forms in proximity of the injection contact. In this high-field domain, the periods of the active region are subject to a voltage drop that causes misalignement of the energy levels and cannot produce gain. However, the remaining periods are still aligned properly. As the bias voltage increases, the high-field domain extends progressively into the whole active region, eventually turning the laser off. Together with the bleaching of the NDR, we interpret the increased dynamic range in sample B as an indication that the thin contact has changed the dynamics of high-field domain formation in the structure, which is responsible for the occurrence of the NDR. It is worth mentioning that, being the responsivity of the pyroelectric detector unknown in the microwave regime, the vertical scale in Figs 1(b) and 2(a–c) shows the raw voltage signal read by the pyroelectric sensor.

The microwave origin of the high-current signal was assessed with a 30 GHz microwave spectrum analyzer, which collected the signal from a dipole antenna placed in front of the cryostat window. Although this did not allow calibration of the measured power, it provides a reliable way to extract the emission frequency. A clear signal was visible on the spectrum analyzer when the QCL was driven in the regime corresponding to strong non-THz emission, as shown in Fig. 2(d), for sample B, at heat-sink temperatures between 250 and 300 K. Whilst this measurement proves the microwave origin of the signal observed at high current densities, the fact that it is present as such high temperatures also suggests that it does not originate from the electron dynamics in the quantized states of the GaAs/AlGaAs heterostructure, but, conversely, it is a bulk phenomenon.

**Figure 3:**
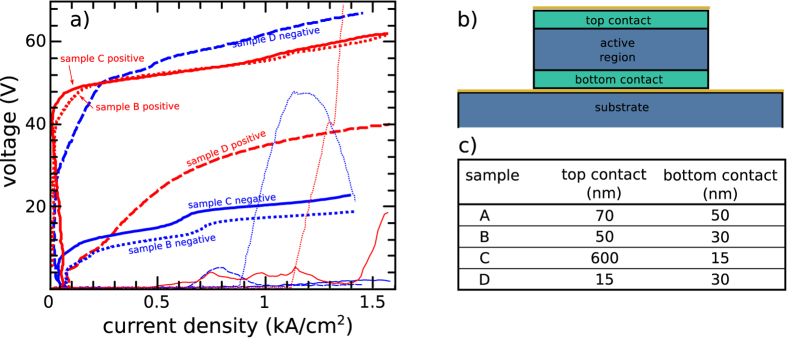
Samples performance. (**a**) IV characteristics of samples B (dotted line), C (solid line) and D (dashed line) in positive (red) and negative (blue) polarity. (**b**) Sketch of the sample cross-section. Positive polarity is defined as positive voltage applied on the top contact. (**c**) Summary of the contact layer thicknesses for all investigated samples.

Upon driving device B under reverse bias (with the negative bias applied to the 50-nm-thick top contact), the prominent threshold voltage V_T_ observed in Fig. 1(b) was not observed. This suggests that the 30-nm-thick highly-doped GaAs bottom contact acts as a diode, preventing electron injection under forward bias, but allowing electrons to flow when injected from the top of the laser. To corroborate this, a further series of samples with an identical active region design, but different contact layer thicknesses, were investigated: sample C had a 600-nm-thick n^+^ top-contact and a 15-nm-thick n^+^ bottom-contact, while sample D had a 15-nm-thick n^+^ top contact and a 30-nm-thick n^+^ bottom contact. The IV characteristics of samples B, C and D under both forward and reverse bias polarities are shown in Fig. 3(a). For clarity, a schematic of the device cross section is shown in Fig. 3(b), and a summary of the contact layer thicknesses in all four devices is shown in Fig. 3(c). All devices showed laser action in both positive and negative polarities, except sample D under positive bias. In all lasing devices, when electrons were injected through a contact layer less than 30 nm thick, a threshold voltage of about 40 V is observed (Fig. 3(a)). This behavior can be explained by assuming that in these samples the n^+^ layer is so thin that it is fully depleted adjacently to the metal contact, and thus the GaAs/AlGaAs heterostructure, which has a two orders of magnitude lower average doping, is also partially depleted. This effectively forms a Schottky barrier with a large threshold voltage. Indeed, bandstructure calculations show that the depletion into the active region of the QCL is ~2.7 μm when the n^+^ layer is only 30 nm thick. In this case, a sizable fraction of the active region is depleted, causing a reduction of optical gain which is in agreement with the observed reduction in the maximum operating temperature in sample B with respect to reference sample A.

### Characterization of the low frequency emission

**Figure 4:**
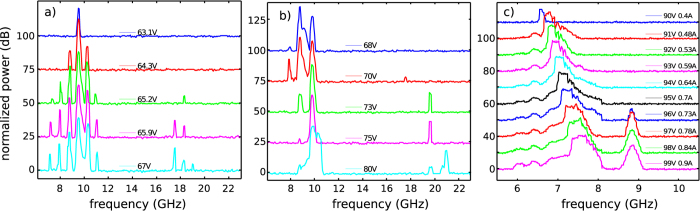
Microwave emission spectra. Microwave emission spectra, measured at different bias voltages and at a heat sink temperature of 70 K, for (**a**) sample B, and (**b**) a nominally identical non-lasing device belonging to the same epitaxial growth (dimensions 760 × 140 μm). The samples were mounted on the cold finger of a helium continuous-flow cryostat and driven in pulsed mode with 200 ns pulses and 10 kHz repetition rate. (**c**) Microwave emission measured at room temperature at various bias conditions for a 360 × 140 μm device, similar to sample B, biased with 400 ns pulses at a 1 kHz repetition rate.

The microwave emission was studied as a function of bias and temperature. Figure 4(a) shows the microwave spectrum of sample B measured at 70 K at different bias voltages. For comparison, the spectra acquired from an identical device in which THz laser action was prevented by damaging the laser facet after repeated stripping of the bonding wires is shown in Fig. 4(b). The data are plotted on a logarithmic scale, normalized to the noise floor of the spectrum analyzer, and offset vertically for clarity. When biased at the microwave emission threshold, a single spectral line is observed in sample B, centered at 9.54 GHz (Fig. 4(a)). As the bias is increased, the spectrum develops a number of sidebands, equally spaced by 780 MHz. At 65.2 V, higher frequency components appear around a central frequency of 18 GHz. In contrast, for the case of the non-lasing device (Fig. 4(b)), the spectrum at threshold (68 V) is characterized by a dominant peak at 8.82 GHz, which hops to 9.9 GHz as the bias increases. A high-frequency component appears at 19.5 GHz when the bias is greater than 73 V. Although the experimental apparatus did not allow calibration of the emitted microwave radiation power, the emission was strong enough to produce signals with a 40 dB signal-to-noise ratio on a −90 dBm noise floor when captured from the free space with a dipole antenna, with the device biased at a 0.2% duty cycle.

The fact that the non-lasing device emits a microwave signal confirms that this phenomenon does not originate from non-linear down-conversion of THz lines in the laser cavity. Furthermore, the evidence that microwave sidebands arise only when the microwave and THz fields coexist, indicates that they arise from a mutual interaction between them, which is expected in the cavity. In fact, it is reasonable to assume that the microwave emission is due to oscillations in the bias current of the QCL, which should, in turn, induce corresponding oscillations in the amplitude of the emitted THz power and the intensity of the intracavity THz field. If the intensity of the intracavity THz field oscillates in time, then a corresponding time variation of the electronic lifetimes of the laser transition is expected, given such lifetimes are dominated by stimulated emission processes. As a result, since electronic lifetimes determine the current flow, a modulation of the THz field induces a modulation of the current and thus of the microwave emission. This effect can be seen as the time-dependent analogue of the situation under stationary conditions, where the electron lifetime reduction induced by the THz photon field leads to a well-known voltage clamping and an increase in the maximum current. In both devices, the −3 dB linewidth of the individual microwave spectral lines is of the order of a few MHz and is due to the 200 ns pulse width used to bias the QCL.

The microwave emission persists up to room temperature in all samples with thinned contacts (e.g. Fig. 4(c)). The emission lies at around 7.5 GHz, and blue shifts upon increasing the applied bias. An additional fixed emission peak at 8.9 GHz becomes visible at a bias of 97 V. The flat-top lineshape of the microwave emission suggests that frequency tuning is taking place during the 400 ns voltage pulse employed to bias the laser, which is likely to be due to the device heating owing to the large peak electrical power dissipated in the device (~100 W). An increase of the lattice temperature of a few tens of Kelvin is expected^24^, leading to the QCL voltage and current ramping during the bias pulse, and this was observed on the oscilloscope trace.

Microwave spectra were observed in all samples where power was detected, but there was no THz component to the emission. This strongly suggests that the large voltage barrier observed in the IV characteristics these samples leads to the microwave emission.

**Figure 5:**
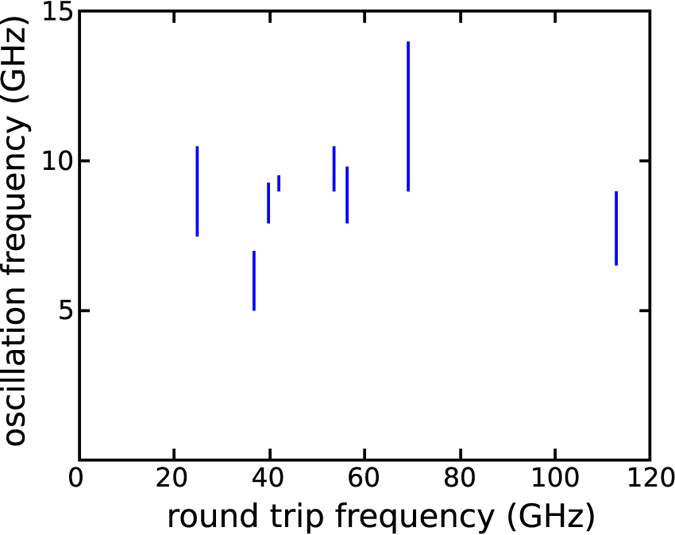
Microwave emission vs round trip. Microwave emission frequencies collected from a set of different samples. The vertical lines mark the microwave emission bandwidth, and their location on the x-axis corresponds to the cavity round-trip frequency of the corresponding QCL device.

To identify any effect associated with the laser cavity, a series of samples with different cavity lengths were investigated. Figure 5 shows the bandwidth of the microwave emission as a function of the cavity round-trip frequency. The lack of correlation in this data suggests that the frequency of the microwave emission is not related to the geometry of the double metal-waveguide. Similarly, no correlation of the microwave emission frequency with the device capacitance could be found, thus excluding inductance-capacitance LC resonances involving the bonding wires. This confirms the attribution of the observed microwave radiation to self-sustained oscillations in the bias current, induced by the presence of a thin n^+^ layer and a large depletion region in proximity to the injection contact.

### Observation of modulated THz emission

**Figure 6:**
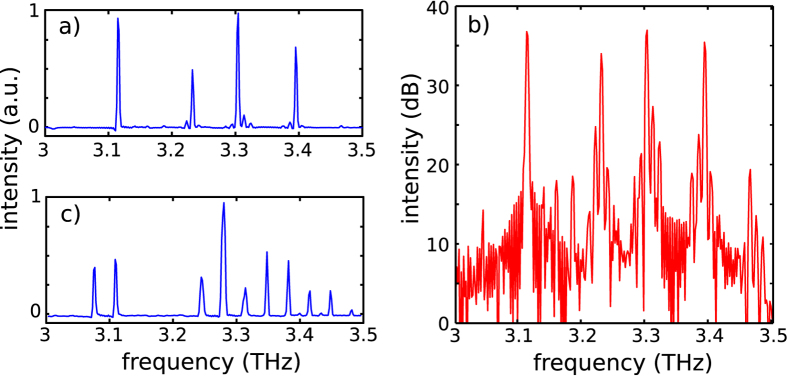
Frequency and amplitude modulation. THz emission spectra of sample B on a (**a**) linear and (**b**) logarithmic scale, measured at a heat sink temperature of 70 K and whilst driving the QCL with a 65 V bias. The spectra were measured while the QCL was operating in pulsed mode (200 ns pulses, 20 kHz repetition rate) using a Fourier transform infrared spectrometer equipped with a helium-cooled Si bolometer, with a spectral resolution of 0.125 cm^−1^, corresponding to 3.7 GHz in rapid-scan mode.

Interaction between the microwave and the THz fields is taking place in the device when the two emission regimes coexist. Figure 6(a,b) show the typical resulting THz emission spectra of sample B on (a) a linear and (b) a logarithmic scale (LIV data in Fig. 2(a–c)). The THz emission spectrum consists of four main modes in the 3.1–3.4 THz range, with the three highest frequency modes surrounded by multiple sidebands spaced by 10 GHz. Since the spacing of the Fabry-Pérot modes in the cavity of sample B is 25 GHz, the observed sidebands cannot be attributed to cavity resonances. However, as the sideband frequency separation matches the microwave emission frequency within the uncertainty of the FTIR resolution, this suggests that the THz emission is frequency modulated, since multiple modulation sidebands cannot be achieved via amplitude modulation only which, conversely, gives rise to two symmetric sidebands around the main THz emission peak for each value of the modulation depth. However, since oscillations in the QCL bias directly translate into oscillations of the THz output power, amplitude modulation is also expected to be present, proportionally to the slope efficiency of each mode. The absence of sidebands around the 3.12 THz mode suggests that the slope efficiency of that specific mode is very small at the expected operating bias. This can be due to the specific gain media design, where population inversion occurs across two different intersubband transitions, which will provide spectral components of the optical gain with a different voltage dependence. For sake of comparison, the spectrum of the reference sample A is plotted in Fig. 6(c). No evidence of modulation sidebands is present, confirming that the RF modulation of the THz emission is a peculiarity of sample B.

### Theoretical modeling

The large voltage threshold V_th_ in the IV curve is a specific feature of all QCL devices for which electron injection occurs through a highly doped contact layer with thickness smaller than 30 nm. Analysis of the optical spectra, however, reveals that sample B operates at the same THz frequency as the reference sample A, meaning that the voltage drop per period in the active core is the same. This leads to the general conclusion that the enhanced voltage threshold in sample B is due to a high-field domain that arises below the injection contact. In addition, the experimental evidence that this enhanced threshold persists, and progressively increases when the temperature is increased from 10 K to room temperature, demonstrates that it cannot originate from the dynamics of electrons within the bound states of the GaAs/AlGaAs heterostructure, because the energy separation of the quantized states is far smaller than k_B_T at room temperature.

In order to develop a simple model for the device, the GaAs/AlGaAs heterostructure was treated as a bulk material, with an average doping corresponding to the average active region *n-type* doping (6.8 × 10^15 ^cm^−3^) and subject to charge depletion owing to the presence of the metallic contact. Energy quantization effects in the thin n^+^ contact layer were neglected. The electrostatics of the contact is then the same as a p^+^/n^+^/n diode, also known as Read diode^25^, with a built-in potential given by approximately half the bandgap of GaAs, as a result of Fermi-level pinning by the GaAs surface states^26^. With these assumptions, the extent of the depletion region and the electric field profile under the contact can be calculated exactly for each bias voltage.

**Figure 7:**
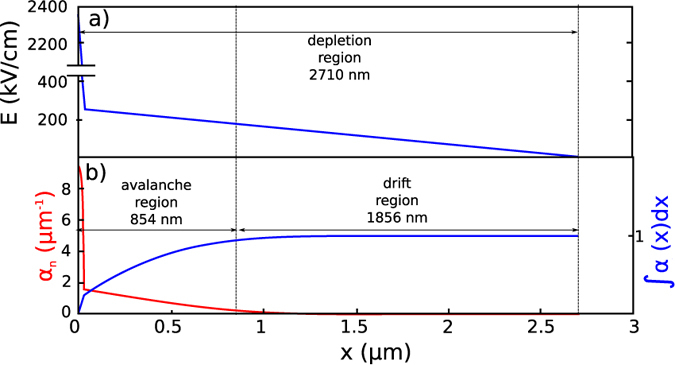
Modelling. (**a**) Static electric field as a function of the distance x from the metallic contact in the depletion region of sample B. (**b**) Impact ionization generation rate (red) and ionization integral (blue). The calculations were performed at a temperature of 20 K and at an applied bias voltage of 37 V, corresponding to the avalanche breakdown voltage.

Figure 7(a) shows the electric field in the active region plotted as a function of the distance x from the metal/GaAs junction, under an operating bias-voltage of 37 V. The depletion region extends 2.7 μm into the bulk, and two distinctive domains characterize the electric field. In the depleted n^+^ region, the electric field at the surface is 2.3 MV/cm and then drops linearly to 260 kV/cm within the thickness of the contact layer (30 nm). In the depleted *n* region, representing the GaAs/AlGaAs heterostructure, the field then decreases from 260 kV/cm to zero. Beyond the depletion region, the field is zero and the conduction band is flat assuming that no current is flowing.

The electric field values calculated in the n^+^ region are high enough to trigger impact ionization of electrons, with an electron generation rates (α_n_) that have an exponential dependency on the electric field strength^27^, and decrease with increasing temperature. The red plot in Fig. 7(b) shows the impact ionization generation rate for electrons (α_n_) plotted as a function of the distance *x* from the metallic junction, while the blue plot represents its integral (ionization integral). The avalanche breakdown voltage of the junction, *V*_*B*_, is defined as the voltage at which the ionization integral reaches unity. For bias voltages up to *V*_*B*_ current does not flow, and thus the flat-band condition can be imposed far away from the contact and a simple model based on stationary electrostatics can be applied. The 37 V bias chosen for the calculations in Fig. 7 corresponds to the calculated avalanche breakdown voltage for sample B. According to ref. 24, in this regime, the depletion region can be divided into an avalanche region, (in which the ionization integral increases to 0.95), and a drift region, in which the avalanche is not present and electrons generated in the avalanche region can drift under the action of the electric field. The injection contact of sample B thus behaves like an IMPATT diode^25^. After the breakdown voltage, current flows from the IMPATT section into the remaining part of the un-depleted QCL active region, where the electric field is not perturbed by the presence of the depletion region. The whole structure can be then regarded as an electric circuit, which includes a QCL in series with an IMPATT diode. Within this model, the resulting IV characteristic corresponds to the QCL IV curve with an additional voltage shift equal to the breakdown voltage of the IMPATT diode, which in turns represents the threshold voltage for current injection in the laser (*V*_*T*_).

The reference sample (A) with a 70 nm injection contact layer does not show a large threshold voltage. In this case, the depletion region is completely contained within the n^+^ contact layer and thus tunneling through the thin triangular Schottky barrier allows current to flow before *V*_*B*_ is reached.

Within this model, the threshold voltage of the Schottky barrier, *V*_*S*_, is defined to be the voltage at which the quantum mechanical transmission coefficient of the Schottky barrier rises above a critical value *K*. The resulting transmission coefficient and its temperature dependence can be calculated using the Wentzel–Kramers–Brillouin (WKB) approximation. It is then assumed that the QCL threshold voltage *V*_*T*_ for each temperature and contact layer thickness is smaller than *V*_*B*_ and *V*_*S*_.

**Figure 8:**
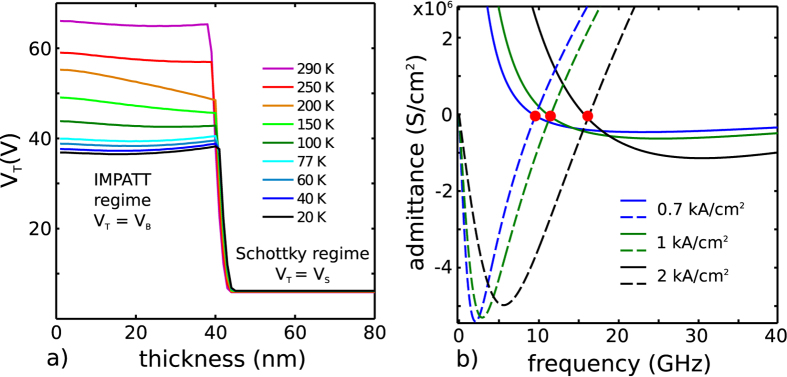
Modelling. (**a**) Injection threshold voltage of sample B calculated as a function of the contact layer thickness and heat sink temperature. (**b**) Real (solid lines) and imaginary (dashed lines) parts of the admittance of the injection diode of sample B calculated for different bias current densities, as a function of frequency. The red dots mark the resonance frequency, defined as the beginning of the negative resistance region.

Figure 8(a) shows the QCL threshold voltage computed according to this model, as a function of the contact layer thickness and temperature. In this model, the temperature affects the electron ionization rates and the transmission through the Schottky barrier. A sharp transition between the avalanche and tunnel regimes is visible on the graph. For large values of the contact layer thickness the depletion region width, and thus the transmission through the Schottky barrier, depends only on the doping of the contact layer. This results in a Schottky threshold *V*_*S*_ that is independent of the contact layer thickness, as is commonly observed in QCLs with conventional contact layers. When the contact layer becomes thinner than this depletion region, the low-doped active region starts to be depleted, giving rise to a large Schottky barrier, whose threshold voltage increases as the contact layer becomes thinner. Eventually, the threshold for tunneling through the triangular barrier becomes larger than the avalanche breakdown voltage, and thus *V*_*T*_ is clamped to *V*_*B*_.

The critical value of the contact layer thickness that separates the IMPATT and Schottky regimes in our model is dictated by the value of *K*, which has been here set to 0.06. When *K* becomes larger, *V*_*S*_ also increases and so does the critical contact layer thickness. We have set the value of *K* based on the knowledge that the Schottky barrier in a conventional double-metal QCL is typically 2–5 V, and on the experimental evidence that a 30 nm contact layer already drives the sample into the IMPATT regime.

The presence of an IMPATT-like section in sample B corroborates the observation of microwave oscillations. Figure 8(b) shows the calculated real (continuous line) and imaginary (dashed line) parts of the admittance of the injection diode as a function of frequency, plotted for different current densities, using the standard expressions for IMPATT diodes. The point where the real and imaginary parts intersect, marked by a red dot in the plot, marks the beginning of the negative differential resistance region, where an oscillatory behavior can arise. By introducing the impact ionization coefficients of GaAs from the literature, resonant frequencies in the 7–20 GHz range are obtained for current densities of 0.7–2 kA/cm^2^, nicely matching qualitatively the experimental data reported in Fig. 4(c). It is worth mentioning that the description of the low temperature microwave oscillation would require a more refined theoretical model that takes into account energy quantization effects.

## Discussion and Conclusions

We have engineered and investigated a number of THz frequency QCLs sharing an identical active region core, but embedded between heavily doped contact layers of different thicknesses (15, 30, 70, 600 nm). We observe that QCLs containing an injection contact layer of 15 nm or 30 nm thickness have an I–V curve characterized by a large threshold voltage that increases with temperature, but these devices still emit THz radiation with emission spectra that are consistent with those of a reference QCL sample with a 70 nm-thick contact layer. We deduce that the alignment of the heterostructure in the bulk is unaffected by the thinning of the contact, and thus the observed overvoltage arises from a depletion region close to the metallic junction which extends into the GaAl/AlGaAs heterostructure and creates a large injection barrier.

Remarkably, the presence of the voltage barrier in the IV curve is always accompanied by intense (Fig. 4) microwave emission, characterized by discrete emission lines in the 5–11 GHz band, with some high frequency peaks around 20 GHz. The microwave oscillations not only occur at low temperature when the QCL is lasing at the designed frequency, but also persist up to room-temperature. This indicates that the microwave oscillation is a bulk-related effect rather than a feature connected with the electron confinement in the heterostructure. In the bias and temperature regimes in which THz and microwave emissions coexist, the THz emission lines show sidebands with a frequency spacing that corresponds to the microwave emission frequency. This implies that intra-cavity frequency and amplitude modulation of THz emission occurs through interaction with the generated microwave radiation.

The observed phenomena have been explained through an analytical model based on the assumption that an IMPATT diode is formed at the injection contact. The model is based on the calculation of the static electric field distribution in the contact region, and the assumption that current injection is governed by a mixed avalanche-tunneling mechanism. Within this model we are able to calculate the threshold voltage of the injection contact and obtain good agreement with the experimental data, including the temperature dependence of the threshold voltage. This confirms that the device operation is dominated by an IMPATT-like injection mechanism. Furthermore, we calculated the microwave impedance of the input IMPATT diode as a function of the frequency. We found that a negative conductance region exists in the 7–20 GHz frequency range, depending on the current density. The observation of tunable, room-temperature, microwave oscillation in the same frequency range provides a further confirmation that an avalanche generation process occurs in the depletion region and is the cause of the observed microwave emission. A quantitative model of the emission frequency would, however, require the development of numerical models that are beyond the scope of the present work.

We propose that our devices can be treated as a series circuit comprising an avalanche diode and a QCL, with the avalanche section providing a negative conductance, that under certain bias conditions triggers microwave oscillations that modulate the THz emission of the QCL. In the devices reported here, the input diode is parasitic and based on the depletion of the QCL active region. The large threshold voltage of the diode prevents the devices from operating in continuous wave, and the oscillation frequency could not be matched with the cavity round-trip time of the QCL. The fabrication of devices where the doping of the depletion region is optimized to increase the value of the avalanche integral in a narrow region close to the contact will, according to our model, increase the resonant frequency of the IMPATT section, whilst leaving a larger portion of the QCL un-depleted. By lowering the threshold voltage, THz QCLs will be capable of operating continuous wave. This would then allow self-mode locking and comb operation of the QCL to be achieved by matching the microwave oscillation frequency to the cavity round trip time of the laser.

## Methods

### Growth and fabrication details

The GaAs/Al_0.15_Ga_0.85_As active region employed in the present work, common to all investigated samples, is based on the three-well design that has shown the highest operating temperature in the literature at THz frequencies^23^, and was grown on a GaAs substrate using molecular beam epitaxy, with an Al_0.5_Ga_0.5_As etch stop layer grown between the active region and the substrate. The contact layers are Si-doped at 5 × 10^18 ^cm^−3^. A Cr/Au 10/500 nm metal layer was thermally evaporated on both the sample wafer and an n^+^ GaAs carrier wafer. After metallization, the wafers where placed together with the Au-coated surfaces facing each other. The stack was then wafer-bonded at 320 °C for 30 minutes at a pressure of 4.5 Mpa. The sample substrate was subsequently lapped down to a 50 μm thickness and then etched down to the etch stop layer using a citric acid solution. After removal of the etch stop layer using hydrofluoric acid, the top contact of the lasers was defined via optical litography and lift-off of a Cr/Au 10/150 nm metallization. Optical lithography and wet etching in H_2_SO_4_:H_2_O_2_:H_2_O 1:1:5 solution was then used to define the laser ridges. Chips containing a number of laser bars were then cleaved and indium soldered onto copper mounts.

### Simulation code

Simulations were performed with custom Matlab scripts. Calculations of the condution band profile and electric field intensity were carried on by implementing standard textbook equations from ref. 24, with the dependency of the impact ionization rates on electric field being taken from ref. 26. The energy dependent Schottky barrier transmission *K(E)* was calculated numerically in the WKB approximation, and the effective transmission coefficient *K* used for the determination of the Schottky barrier threshold was obtained by a thermal average of *K(E).* The admittance of the depletion region was calculating by numerically solving equations from ref. 24.

### Optical and microwave testing

Light-current-voltage measurements were performed in a closed cycle refrigerator equipped with a high-density polyethylene (HDPE) window. Light was collected using a round, 3 mm diameter, pyroelectric detector placed in front of the cryostat window, at 5 mm distance from the sample facet. The detector output was measured by a lock-in amplifier operating at a modulation frequency of 33 Hz. THz spectra were acquired using a Fourier transform infrared spectrometer equipped with a helium-cooled silicon bolometer. The samples were mounted on the cold finger of a helium continuous-flow cryostat equipped with a HDPE window, and light was collected via a 2” f/1 off-axis parabolic. The resolution of the FTIR was 0.125 cm^−1^. Microwave spectra were acquired using an Anritsu microwave spectrum analyzer with 30 GHz bandwidth, by performing full scan measurements in max-hold mode with a 100 kHz resolution bandwidth. The signal was collected using a home-made dipole antenna placed in close proximity of the cryostat window. The length of the dipole antenna, which is inherently broadband, was 6 cm, providing a fundamental resonance frequency of 2.5 GHz.

## Additional Information

**How to cite this article**: Castellano, F. *et al.* Frequency and amplitude modulation of ultra-compact terahertz quantum cascade lasers using an integrated avalanche diode oscillator. *Sci. Rep.* **6**, 23053; doi: 10.1038/srep23053 (2016).
